# Frailty and In-Hospital Outcomes for Management of Cardiogenic Shock without Acute Myocardial Infarction

**DOI:** 10.3390/jcm13072078

**Published:** 2024-04-03

**Authors:** Dae Yong Park, Yasser Jamil, Yousif Ahmad, Theresa Coles, Hayden Barry Bosworth, Nikhil Sikand, Carlos Davila, Golsa Babapour, Abdulla A. Damluji, Sunil V. Rao, Michael G. Nanna, Marc D. Samsky

**Affiliations:** 1 Department of Medicine, Cook County Health, Chicago, IL 60612, USA; 2 Department of Medicine, Yale School of Medicine, New Haven, CT 06510, USA; 3 Section of Cardiovascular Medicine, Yale School of Medicine, New Haven, CT 06510, USA; 4 Department of Population Health Sciences, Duke University School of Medicine, Durham, NC 27710, USA; 5 Department of Medicine, Division of General Internal Medicine, Department of Psychiatry and Behavioral Sciences School of Nursing, Duke University Medical Center, Durham, NC 27701, USA; 6 School of Medicine, Johns Hopkins University, Baltimore, MD 21218, USA; 7 Inova Center of Outcomes Research, Falls Church, VA 22042, USA; 8 NYU Langone Health System, Grossman School of Medicine, New York University, New York, NY 10016, USA

**Keywords:** cardiogenic shock, frailty, non-acute myocardial infarction

## Abstract

(1) **Background**: Cardiogenic shock (CS) is associated with high morbidity and mortality. Frailty and cardiovascular diseases are intertwined, commonly sharing risk factors and exhibiting bidirectional relationships. The relationship of frailty and non-acute myocardial infarction with cardiogenic shock (non-AMI-CS) is poorly described. (2) **Methods**: We retrospectively analyzed the National Inpatient Sample from 2016 to 2020 and identified all hospitalizations for non-AMI-CS. We classified them into frail and non-frail groups according to the hospital frailty risk score cut-off of 5 and compared in-hospital outcomes. (3) **Results**: A total of 503,780 hospitalizations for non-AMI-CS were identified. Most hospitalizations involved frail adults (80.0%). Those with frailty had higher odds of in-hospital mortality (adjusted odds ratio [aOR] 2.11, 95% confidence interval [CI] 2.03–2.20, *p* < 0.001), do-not-resuscitate status, and discharge to a skilled nursing facility compared with those without frailty. They also had higher odds of in-hospital adverse events, such as acute kidney injury, delirium, and longer length of stay. Importantly, non-AMI-CS hospitalizations in the frail group had lower use of mechanical circulatory support but not rates of cardiac transplantation. (4) **Conclusions**: Frailty is highly prevalent among non-AMI-CS hospitalizations. Those accompanied by frailty are often associated with increased rates of morbidity and mortality compared to those without frailty.

## 1. Introduction

Frailty is characterized by increased vulnerability to internal and external stressors and has been demonstrated to be associated with functional limitations and susceptibility to adverse events [1]. The presence of frailty places individuals with cardiovascular diseases at an elevated risk of experiencing complications, longer hospital stays, and major adverse cardiovascular events [2]. As the aging population continues to grow, the anticipated rise in frailty is concerning, posing an increasingly significant global health burden [3,4,5].

Individuals who present with non-acute myocardial infarction (AMI) cardiogenic shock (CS) often suffer from common cardiovascular conditions such as malignant arrhythmias, valvular heart disease, cardiomyopathies, and myocarditis. These underlying chronic comorbidities can independently contribute to reduced physical function, sarcopenia, disability, inflammation, and end-organ dysfunction [6,7]. The association between frailty and CS deserves attention due to the rising prevalence of non-AMI-CS-related hospitalizations, accounting for up to 70% of all CS hospitalizations in certain areas [8,9,10]. Despite this, there has been little investigation of the association between frailty and non-AMI-CS [11,12].

This study aims to investigate the relationship between frailty and outcomes among hospitalizations for non-AMI-CS cases. Our objectives include determining the prevalence of frailty among non-AMI-CS hospitalizations and analyzing its correlations with in-hospital outcomes, with the aim of underscoring the need for systematic frailty assessment in hospitalizations for acute cardiovascular illness.

## 2. Materials and Methods

### 2.1. Data Source

We performed a retrospective cohort study using the National Inpatient Sample (NIS), the largest all-payer inpatient healthcare database in the United States, designed to produce national estimates of inpatient utilization, access, costs, outcomes, and quality [13]. Developed for the Healthcare Cost and Utilization Project (HCUP) and sponsored by the Agency for Healthcare Research and Quality, the NIS collects data from more than 7 million admissions annually and approximates 35 million hospitalizations across 49 participating states when weights are applied. The NIS covers more than 97% of the total population, allowing the study of specific conditions and procedures on a national level. The NIS protects patient confidentiality by excluding state and hospital identifiers, thereby guaranteeing anonymity, and because of this strictly deidentified nature of the database, our study was exempt from the purview of our institutional review board. The NIS is openly available and can be accessed through the public website of the HCUP [13].

### 2.2. Study Population and Covariates

We identified all admissions with CS coded in either primary or secondary diagnoses from the years 2016 to 2020 [14]. Afterward, we excluded patients aged less than 18 years and entries that were missing values for demographics, hospital characteristics, primary payer, median income, day of admission, in-hospital mortality, or length of hospital stay (LOS). We excluded all patients with any diagnosis of AMI. From the remaining dataset, we collected data on demographics (sex, age, race), hospital characteristics (region, bed size, urban location), primary payer, median income, and day of admission (weekday, weekend), which are given in the database. Among all admissions, we detected the presence of multiple comorbidities; listed in Table 1. We also calculated the hospital frailty risk score, a validated measure of clinical frailty, for each admission by bestowing prespecified scores to 109 individual International Classification of Diseases, Tenth Revision, and Clinical Modification codes (Table S1) [15]. A hospital frailty risk score of at least 5 defined frailty, consistent with the definition used by the previous literature [16,17,18]. The hospital frailty risk score has been tested and validated among several studies, demonstrating a correlation between mortality, functional impairment, and quality-of-life outcomes [15,19]. All the comorbidities and procedural data we used were established based on the International Classification of Diseases, Tenth Revision, Clinical Modification and International Classification of Diseases, Tenth Revision, Procedural Coding System codes. These codes are listed in Table S2.

### 2.3. Study Outcomes

We set our primary outcome as in-hospital mortality. Secondary outcomes consisted of do-not-resuscitate status (DNR), palliative care consult, disposition to a skilled nursing facility, use of mechanical circulatory support (MCS), heart transplant, intracranial hemorrhage, gastrointestinal hemorrhage, acute kidney injury, delirium, LOS, and total hospital cost. Using an intra-aortic balloon pump, percutaneous left ventricular assist device, durable left ventricular assist device, or extracorporeal membranous oxygenation defines MCS. We calculated total hospital cost by multiplying the given total hospital charge with the cost-to-charge ratios available in cost-to-charge files on an ancillary website of HCUP [20].

### 2.4. Statistical Analysis

We applied hospital-level discharge weights to all entries when performing all statistical analyses to produce national estimates. We used the chi-square and Kruskal–Wallis H tests to compare categorical and continuous covariates in the baseline characteristics, respectively. To select covariates included in statistical adjustment, we first examined all baseline characteristics in a correlation matrix to confirm that no two covariates had a Pearson correlation coefficient above 0.80 (Table S3). Second, we confirmed that all covariates had a variance inflation factor below 3 and a tolerance value above 0.1. Third, we double-checked the absence of multicollinearity in an eigensystem analysis of covariance. After resolving multicollinearity, we inserted all covariates in a multivariable logistic regression model comparing non-AMI-CS admissions with and without frailty and identified significant covariates by stepwise selection. Age, sex, smoking, diabetes mellitus, hyperlipidemia, obesity, heart failure, chronic ischemic heart disease, valvular heart disease, previous percutaneous coronary intervention, previous coronary artery bypass grafting, previous stroke, previous pacemaker, chronic obstructive pulmonary disease, pulmonary hypertension, end-stage renal disease, deficiency anemia, malnutrition, major depression, and weekend admission were used to adjust all statistical models.

We used univariable and multivariable logistic regression to produce crude odds ratios and adjust odds ratios (aOR), respectively, to compare binary outcomes. We used linear regression when comparing secondary outcomes, such as LOS and total hospital cost. We performed a subgroup analysis stratified to younger (age 18–64) and older (age ≥ 65) adults. All statistical tests were two-sided, and *p*-values < 0.05 were considered significant. Data curation and all statistical analyses were performed using SAS, version 9.4 (SAS Institute, Cary, NC, USA). Figure production was assisted by R version 4.0.5 (R Foundation for Statistical Computing, Vienna, Austria).

## 3. Results

**Figure 1:**
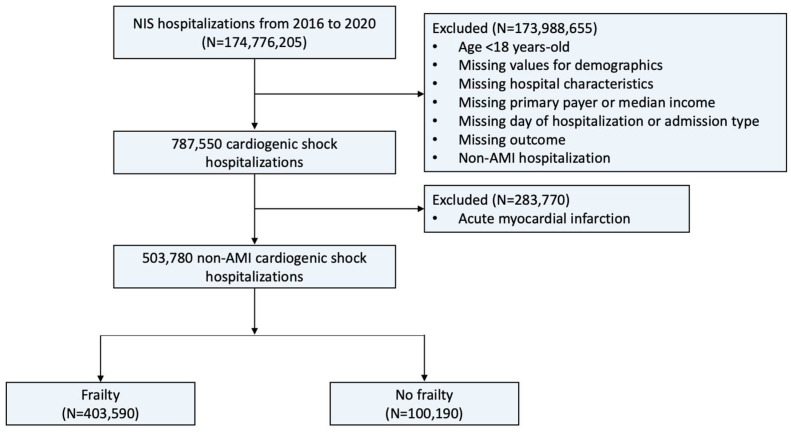
Flowchart of this study. **Description:** The flowchart illustrates the patient selection process used in this study. **Abbreviations:** AMI, acute myocardial infarction; NIS, National Inpatient Sample.

A total of 503,780 admissions for non-AMI-CS were identified (Figure 1). Most (80.1%) occurred in the frail, while a minority (19.9%) occurred in the non-frail. The frail group had a higher median age compared with the non-frail group (68 vs. 65, *p* < 0.001) (Table 1). The percentage of hospitalizations for patients classified as Black was greater in the frail group (19.8% vs. 17.7%, *p* < 0.001). The frail group had a higher prevalence of diabetes mellitus, heart failure, chronic ischemic heart disease, atrial fibrillation, previous stroke, chronic kidney disease, malnutrition, and dementia but a lower prevalence of hypertension.

**Table 1:** Baseline characteristics of non-AMI-CS admissions with and without frailty.

	Frailty (+)	Frailty (−)	*p*-Value
**Number of admissions**	403,590	100,190	
**Male sex (%)**	61.1	63.3	<0.001
**Age, mean (Q1–Q3), years**	68 (57–77)	65 (54–73)	<0.001
**Race (%)**			<0.001
White	64.9	67.1	
Black	19.8	17.7	
Hispanic	8.7	8.6	
Asian	3.0	3.0	
AI/AN	0.6	0.7	
Other	3.0	3.0	
**Comorbidities (%)**			
Smoking	33.3	33.6	0.442
Hypertension	11.8	20.2	<0.001
Diabetes mellitus	38.6	34.5	<0.001
Hyperlipidemia	39.2	45.2	<0.001
Obesity	18.2	18.1	0.761
Heart failure	71.7	63.2	<0.001
Chronic ischemic heart disease	17.5	15.6	<0.001
Atrial fibrillation	45.1	38.5	<0.001
Valvular heart disease	13.0	15.4	<0.001
Peripheral artery disease	6.9	6.1	<0.001
Previous PCI	0.9	1.3	<0.001
Previous CABG	7.9	8.9	<0.001
Previous stroke	10.0	7.3	<0.001
Previous pacemaker	3.5	3.8	0.087
COPD	24.9	20.1	<0.001
Pulmonary hypertension	20.8	19.6	<0.001
Chronic kidney disease	47.7	22.5	<0.001
End-stage renal disease	9.8	4.0	<0.001
Liver cirrhosis	8.0	5.9	<0.001
History of malignancy	7.3	8.1	<0.001
Deficiency anemia	7.4	5.9	<0.001
Malnutrition	17.3	8.8	<0.001
Dementia	6.6	0.9	<0.001
Major depression	0.9	0.8	0.173
HFRS, median (Q1-Q3)	8.8 (7.0–11.3)	3.4 (2.2–4.2)	<0.001
**Hospital characteristics (%)**			
**Hospital region**			<0.001
Northwest	16.5	19.8	
Midwest	22.8	19.5	
South	40.1	41.5	
West	20.7	19.2	
**Hospital bed size**			<0.001
Small	12.9	11.9	
Medium	23.6	22.2	
Large	63.5	65.9	
**Urban location**			<0.001
Rural	3.5	3.7	
Urban non-teaching	14.5	13.4	
Urban teaching	82.0	82.9	
**Primary payer (%)**			<0.001
Medicare	63.5	53.6	
Medicaid	13.1	14.1	
Private insurance	17.9	25.5	
Self-pay	2.9	3.5	
No charge	0.2	0.3	
Others	2.6	3.1	
**Median income (%)**			0.002
Quartile 1	31.5	30.2	
Quartile 2	26.1	26.9	
Quartile 3	23.2	23.4	
Quartile 4	19.1	19.5	
**Day of admission (%)**			<0.001
Weekday	77.2	81.9	
Weekend	22.8	18.1	

**Abbreviations:** AI/AN, American Indian/Alaska Native; AMI, acute myocardial infarction; CABG, coronary artery bypass graft; COPD, chronic obstructive pulmonary disease; CS, cardiogenic shock; HFRS, hospital frailty risk score; PCI, percutaneous coronary intervention; Q, quartile.

**Figure 2:**
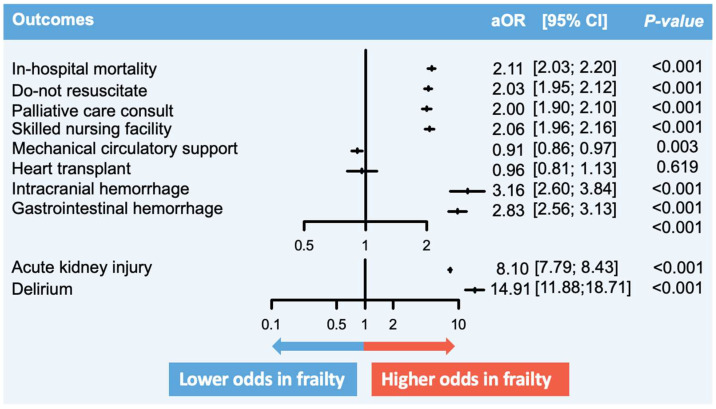
Comparison of in-hospital outcomes between non-AMI-CS in frailty versus no frailty. **Description:** The figure summarizes the key findings of this study. The vertical lines represent the aOR, while the perpendicular horizontal lines represent the 95% CI. aOR > 1 signifies that the odds of the particular outcome are higher in AMI-CS hospitalizations with frailty, and vice versa. **Abbreviations:** AMI, acute myocardial infarction; aOR, adjusted odds ratio; CI, confidence interval; CS, cardiogenic shock.

In admissions for non-AMI-CS, in-hospital death occurred in 35.1% of the frail group compared to 20.4% in the non-frail group (Table 2). Frailty was associated with significantly higher odds of in-hospital mortality (aOR 2.11, 95% confidence interval [CI] 2.03–2.20, *p* < 0.001). The presence of frailty was also associated with higher odds of receiving a palliative care consultation (aOR 2.00, 95% CI 1.90–2.10, *p* < 0.001) and having a DNR order placed (aOR 2.03, 95% CI 1.95–2.12, *p* < 0.001). Frailty had higher odds of disposition to a skilled nursing facility (aOR 2.06, 95% CI 1.96–2.16, *p* < 0.001). It had lower odds of being managed with MCS (aOR 0.91, 95% CI 0.86–0.97, *p* = 0.003). No significant difference in the odds of heart transplant was observed (aOR 0.96, 95% CI 0.81–1.13, *p* = 0.619). Frailty was associated with higher in-hospital morbidities, including intracerebral hemorrhage, gastrointestinal hemorrhage, acute kidney injury, and delirium. The LOS and total hospital cost were significantly higher in the frail. No significant difference was seen between univariable and multivariable models (Figure 2).

Stratification to age cut-off of 65 years showed that 216,375 (42.9%) non-AMI-CS occurred in younger adults, while 287,405 (57.1%) occurred in older adults. Although the prevalence of frailty was high regardless of age group, it was lower in younger adults compared with older adults (76.9% vs. 82.5%, *p* < 0.001). The results were largely similar in younger adults, except for the odds of MCS, which were not different between the frail and non-frail (aOR 1.05, 95% CI 0.97–1.12, *p* < 0.001) (Table S3). Similar results were seen in the older population, in which frailty was associated with higher odds of in-hospital mortality, DNR, palliative care consult, skilled nursing facility, heart transplant, intracerebral hemorrhage, gastrointestinal hemorrhage, acute kidney injury, and delirium, but lower odds of MCS.

## 4. Discussion

In this national retrospective analysis, we aimed to explore the association between frailty and non-AMI-CS hospitalizations. Our findings highlight an 80% prevalence of frailty, coupled with in-hospital morbidity and mortality. The frail subset also demonstrated a clear propensity for multi-morbidity (≥2 chronic illnesses). Importantly, frailty was linked to a reduced likelihood of cardiac interventions, notably MCS, but with no impact on cardiac transplantation rates.

Frailty’s impact on prognostic outcomes has been extensively explored in acute cardiovascular diseases such as AMI and decompensated heart failure. However, our understanding of frailty in the non-AMI-CS population remains limited [12]. This topic is important due to the high rates of frailty in individuals with cardiovascular diseases. The rates vary widely, ranging from 12.6% to 70%. These variations are likely attributable to differences in diagnostic tools, the absence of frailty assessment, and variations in acute cardiovascular conditions and baseline characteristics. Notably, this current study revealed a staggering 80% prevalence of frailty in non-AMI-CS [21,22]. This elevated prevalence and the associated heightened mortality risk emphasizes the imperative for standardized frailty assessments, facilitating tailored interventions and care discussions in clinical practice and patient-centered decision making [1,23].

It comes as no surprise that frailty was associated with a higher incidence of in-hospital complications and increased mortality, similar to prior studies [2,21,22,24]. This propensity is likely related to the presence of multi-morbidity, including diabetes, hypertension, heart failure, coronary artery disease, peripheral artery disease, and valvular dysfunction [25]. These comorbid conditions can potentially compromise physical activity and cognitive function. Consequently, such patients are rendered more vulnerable to stressors, with their capacity to rebound from these stressors potentially impaired [26,27]. In addition, frailty is intricately linked to hemodynamic alterations, subclinical vascular modifications, and autonomic dysfunction, further complicating the intricate hemodynamic balance and potentially altering compensatory mechanisms during acute decompensation as in CS [28,29]. Moreover, frail individuals often have diminished independence, often present late, and are more likely to be managed conservatively [30,31,32]. Lastly, there are higher in-hospital complications, such as delirium, which could prolong and lead to long-term sequelae (physical and psychological) [33,34]. Therefore, frail hospitalizations are often complicated with higher in-hospital mortality and morbidity, raising the question of whether frailty hospitalizations should be approached differently.

This study highlights that frailty is not exclusive to octogenarians and can manifest in a younger subset of patients. The mean age for those hospitalized with frailty was 66.1 years, necessitating age differentiation from frailty [35,36]. Furthermore, in line with existing evidence, frail adults are less likely to receive invasive interventions and evidence-based strategies, including MCS and intensive medical therapy [37,38]. For instance, in a post hoc analysis from the GUIDE-IT trial, those with a high frailty burden and heart failure were less likely to achieve goal-directed medical therapy [39]. Despite ongoing uncertainty surrounding the benefits of intensive strategies in this population, compounded by potential risks and complications, identifying a specific subset of the frail who may benefit from more intensive approaches remains an unmet challenge. Developing a targeted approach for using MCS in this patient population may result in improved clinical outcomes and minimize potential complications. Alternatively, identifying patients who are highly unlikely to benefit from MCS under any circumstance could lead providers to have earlier discussions about the goals of care with patients [40,41]. Lastly, this study found no differences in cardiac transplant rates between frail and non-frail groups. This is likely because frail individuals are prioritized in the national emergency priority system due to their critical condition. Observational data showed shorter waiting times for frail patients (0.6 years vs. 0.2 years) [42].

Several limitations should be acknowledged in this retrospective study. This observational, administrative data analysis relies on diagnosis codes and the associated coding errors related to institutional practice or individual bias. Reliance on administrative data lacks clinical information related to the severity and duration of preceding frailty and the CS state. Moreover, although AMI was excluded, the etiology of non-AMI-CS was unknown due to the limitations of the database. Additionally, the severity of comorbidities such as diabetes and hypertension are unknown, which could also impact frailty differently. While hospital frailty risk scores have been validated and have demonstrated a fair to moderate overlap with the Fried and Rockwood scales, it is important to note that comorbidities can influence the score rather than frailty alone. Each entry provided in the NIS consists of hospitalizations and not patients, so our findings should be interpreted at the hospitalization level and after considering that the same patient can be included more than once, given the absence of patient-level identifiers. Lastly, the specifics of shared decision making regarding invasive or conservative therapy are unknown, posing a potential risk of selection bias.

## 5. Conclusions

In this nationally representative sample of hospitalizations for non-AMI-CS, frailty emerged as a noteworthy factor associated with elevated mortality rates and in-hospital complications. Hospitalizations characterized by frailty were less likely to undergo invasive interventions. Further studies are needed to investigate the potential benefits of interventions targeting frailty in this patient population.

## Figures and Tables

**Table 2:** Comparison of outcomes in non-AMI-CS with and without frailty.

Outcome	Frailty (+)	Frailty (−)	Crude Odds Ratio	*p*-Value	Adjusted Odds Ratio ^a^	*p*-Value
In-hospital mortality (%)	35.1	20.4	2.11 (2.03–2.20)	<0.001	2.11 (2.03–2.20)	<0.001
Do not resuscitate (%)	30.2	16.2	2.24 (2.14–2.33)	<0.001	2.03 (1.95–2.12)	<0.001
Palliative care consultation (%)	23.3	12.1	2.21 (2.10–2.31)	<0.001	2.00 (1.90–2.10)	<0.001
Skilled nursing facility (%)	26.2	12.9	2.39 (2.28–2.51)	<0.001	2.06 (1.96–2.16)	<0.001
MCS (%)	11.2	13.4	0.82 (0.77–0.87)	<0.001	0.91 (0.86–0.97)	0.003
Heart transplant (%)	1.3	1.5	0.88 (0.75–1.04)	0.125	0.96 (0.81–1.13)	0.619
Intracranial hemorrhage (%)	1.7	0.6	2.98 (2.46–3.61)	<0.001	3.16 (2.60–3.84)	<0.001
Gastrointestinal hemorrhage (%)	6.8	2.3	3.05 (2.76–3.37)	<0.001	2.83 (2.56–3.13)	<0.001
Acute kidney injury (%)	73.1	29.2	6.59 (6.35–6.84)	<0.001	8.10 (7.79–8.43)	<0.001
Delirium (%)	5.8	0.4	15.92 (12.69–19.97)	<0.001	14.91 (11.88–18.71)	<0.001
Length of stay (days ± SD)	13.1 ± 15.6	8.4 ± 10.1	4.77 (4.55–5.00) ^b^	<0.001	4.12 (3.90–4.33) ^c^	<0.001
Total hospital cost (USD ± SD)	60,777 ± 88,028	42,792 ± 59,142	17,985 (16,699–19,270) ^b^	<0.001	17,277 (16,031–18,522) ^c^	<0.001

^a^ Adjusted for age, sex, smoking, diabetes mellitus, hyperlipidemia, obesity, heart failure, chronic ischemic heart disease, valvular heart disease, previous PCI, previous CABG, previous stroke, previous pacemaker, chronic obstructive pulmonary disease, pulmonary hypertension, end-stage renal disease, deficiency anemia, malnutrition, major depression, and weekend admission. ^b^ Crude mean difference with 95% confidence interval. ^c^ Adjusted mean difference with 95% confidence interval. **Abbreviations:** AMI, acute myocardial infarction; CABG, coronary artery bypass graft; CS, cardiogenic shock; MCS, mechanical circulatory support; SD, standard deviation.

## Data Availability

The NIS is openly available and can be accessed through the public website of the HCUP.

## Supplementary Materials

The following supporting information can be downloaded at: https://www.mdpi.com/article/10.3390/jcm13072078/s1, Table S1: ICD-10-CM codes used to calculate the Hospital Frailty Risk Score; Table S2: List of the ICD-10-CM codes used, Table S3: Subgroup analysis according to younger (age < 65 years) and older (≥65 years) patients.
