# Supplementary material for: Frailty and In-Hospital Outcomes for Management of Cardiogenic Shock without Acute Myocardial Infarction
Source: J Clin Med. 2024 Apr 3;13(7):2078. doi: 10.3390/jcm13072078 (PMC11012362; doi:10.3390/jcm13072078)
Supplement: Supplementary file 1 [file jcm-13-02078-s001.zip › jcm-2854258-supplementary.pdf]

Table S1. ICD-10-CM codes used to calculate the Hospital Frailty Risk Score

ICD-10-CM	Description	Awarded Points
F00	Dementia in Alzheimer's disease	7.1
G81	Hemiplegia	4.4
G30	Alzheimer's disease	4.0
I69	Sequelae of cerebrovascular disease	3.7
R29	Other symptoms and signs involving the nervous and musculoskeletal systems	3.6
N39	Other disorders of urinary system	3.2
F05	Delirium, not induced by alcohol and other psychoactive substances	3.2
W19	Unspecified fall	3.2
S00	Superficial injury of head	3.2
R31	Unspecified hematuria	3.0
B96	Other bacterial agents as the cause of diseases classified to other chapters	2.9
R41	Other symptoms and signs involving cognitive functions and awareness	2.7
R26	Abnormalities of gait and mobility	2.6
I67	Other cerebrovascular diseases	2.6
R56	Convulsions, not elsewhere classified	2.6
R40	Somnolence, stupor, and coma	2.5
T83	Complications of genitourinary prosthetic devices, implants, and grafts	2.4
S06	Intracranial injury	2.4
S42	Fracture of shoulder and upper arm	2.3
E87	Other disorders of fluid, electrolyte, and acid-base balance	2.3
M25	Other joint disorders, not elsewhere classified	2.3
E86	Volume depletion	2.3
R54	Senility	2.2
Z50	Care involving use of rehabilitation procedures	2.1
F03	Unspecified dementia	2.1
W18	Other fall on same level	2.1
Z75	Problems related to medical facilities and other healthcare	2.0
F01	Vascular dementia	2.0
S80	Superficial injury of lower leg	2.0
L03	Cellulitis	2.0
H54	Blindness and low vision	1.9
E53	Deficiency of other B group vitamins	1.9
Z60	Problems related to social environment	1.8
G20	Parkinson's disease	1.8
R55	Syncope and collapse	1.8
S22	Fracture of rib(s), sternum, and thoracic spine	1.8
K59	Other functional intestinal disorders	1.8
N17	Acute renal failure	1.8
L89	Decubitus ulcer	1.7
Z22	Carrier of infectious disease	1.7
B95	Streptococcus and staphylococcus as the causes of diseases classified to other chapters	1.7
L97	Ulcer of lower limb, not elsewhere classified	1.6
R44	Other symptoms and signs involving general sensations and perceptions	1.6
K26	Duodenal ulcer	1.6
I95	Hypotension	1.6
N19	Unspecified renal failure	1.6
A41	Other septicemia	1.6
Z87	Personal history of other diseases and conditions	1.5
J96	Respiratory failure, not elsewhere classified	1.5
X59	Exposure to unspecified factor	1.5
M19	Other arthrosis	1.5
G40	Epilepsy	1.5
M81	Osteoporosis without pathological fracture	1.4
S72	Fracture of femur	1.4
S32	Fracture of lumbar spine and pelvis	1.4
E16	Other disorders of pancreatic internal secretion	1.4
R94	Abnormal results of function studies	1.4
N18	Chronic renal failure	1.4
R33	Retention of urine	1.3
R69	Unknown and unspecified causes of morbidity	1.3

N28	Other disorders of kidney and ureter, not elsewhere classified	1.3
R32	Unspecified urinary incontinence	1.2
G31	Other degenerative diseases of nervous system, not elsewhere classified	1.2
Y95	Nosocomial condition	1.2
S09	Other and unspecified injuries of head	1.2
R45	Symptoms and signs involving emotional state	1.2
G45	Transient cerebral ischemic attacks and related symptoms	1.2
Z74	Problems related to care-provider dependency	1.1
M79	Other soft tissue disorders, not elsewhere classified	1.1
W06	Fall involving bed	1.1
S01	Open wound of head	1.1
A04	Other bacterial intestinal infections	1.1
A09	Diarrhea and gastroenteritis of presume infectious origin	1.1
J18	Pneumonia, organism unspecified	1.1
J69	Pneumonitis due to solids and liquids	1.0
R47	Speech disturbances, not elsewhere classified	1.0
E55	Vitamin D deficiency	1.0
Z93	Artificial opening status	1.0
R02	Gangrene, not elsewhere classified	1.0
R63	Symptoms and signs concerning food and fluid intake	0.9
H91	Other hearing loss	0.9
W10	Fall on and from stairs and steps	0.9
W01	Fall on same level from slipping, tripping, and stumbling	0.9
E05	Thyrotoxicosis	0.9
M41	Scoliosis	0.9
R13	Dysphagia	0.8
Z99	Dependence on enabling machines and deices	0.8
U80	Agent resistant to penicillin and related antibiotics	0.8
M80	Osteoporosis with pathological fracture	0.8
K92	Other diseases of digestive system	0.8
I63	Cerebral infarction	0.8
N20	Calculus of kidney and ureter	0.7
F10	Mental and behavioral disorders due to use of alcohol	0.7
Y84	Other medical procedures as the cause of abnormal reaction of the patient	0.7
R00	Abnormalities of heartbeat	0.7
J22	Unspecified acute lower respiratory infection	0.7
Z73	Problems related to life-management difficulty	0.6
R79	Other abnormal findings of blood chemistry	0.6
Z91	Personal history of risk-factors, not elsewhere classified	0.5
S51	Open wound of forearm	0.5
F32	Depressive episode	0.5
M48	Spinal stenosis	0.5
E83	Disorders of mineral metabolism	0.4
M15	Polyarthrosis	0.4
D64	Other anemias	0.4
L08	Other local infections of skin and subcutaneous tissue	0.4
R11	Nausea and vomiting	0.3
K52	Other noninfective gastroenteritis and colitis	0.3
R50	Fever of unknown origin	0.1

Table S2. List of the ICD-10-CM codes used

Diagnosis	ICD-10 Code
Acute myocardial infarction	I21.0, I21.1, I21.2, I21.3, I21.4, I21.9, I22.0, I22.1, I22.2, I22.8, I22.9
Cardiogenic shock	R57.0
Smoking	F17, T65, Z72.0, O99.33, Z87.891
Hypertension	I10
Diabetes mellitus	E08, E10, E11, E13
Hyperlipidemia	E78
Obesity	E66
Heart failure	I09.81, I11.0, I13.0, I31.2, I50
Chronic ischemic heart disease	I25
Atrial fibrillation	I48.0, I48.1, I48.2, I48.91
Valvular heart disease	I34, I35, I36, I37
Peripheral artery disease	I70
Previous PCI	Z98.61
Previous CABG	Z95.1
Previous stroke	I69, Z86.73
Previous pacemaker	Z95.0
Chronic obstructive pulmonary disease	J41, J42, J43, J44
Pulmonary hypertension	I27.0, I27.2
Chronic kidney disease	N18
End-stage renal disease	N18.6
Liver cirrhosis	K70.2, K70.3, K71.7, K74, K76.1, P78.81, E83.110
History of malignancy	Z85
Deficiency anemia	D50, D51, D52, D53
Malnutrition	E43, E44, E46
Major depression	F33
Palliative care consult	Z51.5, Z71.89
Do not resuscitate	Z66
Coronary revascularization*	027034, 027035, 027036, 027037, 027044, 027045, 027046, 027047, 027134, 027135, 027136, 027137, 027144, 027145, 027146, 027147, 027234, 027235, 027236, 027237, 027244, 027245, 027246, 027247, 027334, 027335, 027336, 027337, 027344, 027345, 027346, 027347, 02703D, 02703E, 02703F, 02703G, 02704D, 02704E, 02704F, 02704G, 02713D, 02713E, 02713F, 02713G, 02714D, 02714E, 02714F, 02714G, 02723D, 02723E, 02723F, 02723G, 02724D, 02724E, 02724F, 02724G, 02733D, 02733E, 02733F, 02733G, 02734D, 02734E, 02734F, 02734G, 02703Z, 02704Z, 02713Z, 02714Z, 02723Z, 02724Z, 02733Z, 02734Z, 02100, 02110, 02120, 02130
Mechanical circulatory support*	5A02110, 5A02210, 5A02116, 5A0211D, 5A02216, 5A0221D, 02HA3, 02HA4, 02HA0QZ, 5A15
Heart transplant*	02YA0Z0, 02YA0Z1, 02YA0Z2
Intracranial hemorrhage	I60, I61, I62, S06.3, S06.4, S06.5, S06.6
Gastrointestinal hemorrhage	I85.01, I85.11, K25.0, K25.2, K25.4, K25.6, K26.0, K26.2, K26.4, K26.6, K27.0, K27.2, K27.4, K27.6, K28.0, K28.2, K28.4, K28.6, K29.01, K29.21, K29.31, K29.41, K29.51, K29.61, K29.71, K29.81, K29.91, K62.5, K92.0, K92.1, K92.2
Acute kidney injury	N17
Delirium	F05, R41.0, R41.82

*ICD-10-PCS codes

Abbreviations: CABG, coronary artery bypass graft; ECMO, extracorporeal membranous oxygenation; LVAD, left ventricular assist device; ICD-10-CM, International Classification of Diseases, Tenth Revision, Clinical Modification; ICD-10-PCS, International Classification of Diseases, Tenth Revision, Procedure Coding System; PCI, percutaneous coronary intervention

Table S3. Pearson’s correlation to screen for collinearity

	AGE	SEX	RACE	smoke	HTN	DM	HLD	OBESITY	HF	CIHD	AF	VHD	PAD	PPCI	PCABG	PS	PP	COPD	PHTN	ckd	ESRD	LC	HMALIG	DA	MAL	DEM	MDD	AWEEKEND
AGE<br>Age in years at admission	1.00000	0.06209<br><.0001	-0.11032<br><.0001	-0.06886<br><.0001	0.03826<br><.0001	0.09087<br><.0001	0.21367<br><.0001	-0.11612<br><.0001	0.08614<br><.0001	0.11595<br><.0001	0.26692<br><.0001	0.05392<br><.0001	0.09453<br><.0001	0.02631<br><.0001	0.12536<br><.0001	0.06292<br><.0001	0.10851<br><.0001	0.12228<br><.0001	0.00488<br>0.1214	0.16360<br><.0001	-0.00917<br>0.0036	-0.07838<br><.0001	0.13331<br><.0001	-0.00571<br>0.0699	0.01463<br><.0001	0.22282<br><.0001	-0.03264<br><.0001	-0.00133<br>0.6724
SEX	0.06209<br><.0001	1.00000	0.00338<br>0.2830	-0.09353<br><.0001	0.03801<br><.0001	-0.01906<br><.0001	-0.03622<br><.0001	0.03790<br><.0001	-0.04384<br><.0001	-0.00768<br>0.0147	-0.04144<br><.0001	0.00559<br>0.0759	-0.01462<br><.0001	-0.02150<br><.0001	-0.09134<br><.0001	0.02028<br><.0001	0.00639<br>0.0424	0.00457<br>0.1469	0.03893<br><.0001	-0.07299<br><.0001	-0.00335<br>0.2874	-0.04128<br><.0001	0.02721<br><.0001	0.02714<br><.0001	0.00172<br>0.5849	0.05301<br><.0001	0.02745<br><.0001	0.01080<br>0.0006
RACE<br>Race (uniform)	-0.11032<br><.0001	0.00338<br>0.2830	1.00000	-0.06719<br><.0001	-0.01933<br><.0001	0.06426<br><.0001	-0.03458<br><.0001	-0.01567<br><.0001	0.00019<br>0.9524	-0.04582<br><.0001	-0.06568<br><.0001	-0.02267<br><.0001	-0.02691<br><.0001	-0.00358<br>0.2560	-0.02949<br><.0001	0.01275<br><.0001	-0.01568<br><.0001	-0.08273<br><.0001	0.01363<br><.0001	0.03716<br><.0001	0.07852<br><.0001	0.02866<br><.0001	-0.05469<br><.0001	0.01813<br><.0001	0.01644<br><.0001	0.00311<br>0.3236	-0.01610<br><.0001	0.00939<br>0.0029
smoke	-0.06886<br><.0001	-0.09353<br><.0001	-0.06719<br><.0001	1.00000	0.05109<br><.0001	-0.02561<br><.0001	0.08299<br><.0001	0.00736<br>0.0195	-0.01199<br>0.0001	0.00058<br>0.8534	-0.01932<br><.0001	0.02711<br><.0001	0.05553<br><.0001	0.03247<br><.0001	0.07794<br><.0001	0.04692<br><.0001	0.02189<br><.0001	0.20585<br><.0001	-0.00408<br>0.1949	-0.03668<br><.0001	-0.06161<br><.0001	0.01923<br><.0001	0.06047<br><.0001	0.00802<br>0.0109	-0.06718<br><.0001	-0.03832<br><.0001	0.00009<br>0.9775	-0.00142<br>0.6513
HTN	0.03826<br><.0001	0.03801<br><.0001	-0.01933<br><.0001	0.05109<br><.0001	1.00000	-0.02795<br><.0001	0.08656<br><.0001	0.01702<br><.0001	-0.42217<br><.0001	-0.03097<br><.0001	-0.05557<br><.0001	-0.02372<br><.0001	0.00252<br>0.4235	-0.00218<br>0.4894	-0.02170<br><.0001	0.00532<br>0.0913	-0.01214<br>0.0001	-0.01994<br><.0001	-0.09358<br><.0001	-0.32162<br><.0001	-0.11655<br><.0001	-0.05894<br><.0001	0.02963<br><.0001	-0.02540<br><.0001	-0.05817<br><.0001	0.01699<br><.0001	-0.00243<br>0.4409	-0.00573<br>0.0687
DM	0.09087<br><.0001	-0.01906<br><.0001	0.06426<br><.0001	-0.02561<br><.0001	-0.02795<br><.0001	1.00000	0.19844<br><.0001	0.16188<br><.0001	0.09702<br><.0001	0.02360<br><.0001	0.02945<br><.0001	-0.01703<br><.0001	-0.00941<br>0.0028	0.02017<br><.0001	0.07429<br><.0001	0.04203<br><.0001	0.00764<br>0.0153	0.03237<br><.0001	0.02306<br><.0001	0.19921<br><.0001	0.11999<br><.0001	-0.00740<br>0.0188	-0.01633<br><.0001	0.01647<br><.0001	-0.05579<br><.0001	0.01701<br><.0001	-0.00713<br>0.0237	-0.00827<br>0.0087
HLD	0.21367<br><.0001	-0.03622<br><.0001	-0.03458<br><.0001	0.08299<br><.0001	0.08656<br><.0001	0.19844<br><.0001	1.00000	0.08858<br><.0001	0.06222<br><.0001	0.07380<br><.0001	0.08730<br><.0001	0.06390<br><.0001	0.07225<br><.0001	0.04839<br><.0001	0.13172<br><.0001	0.07491<br><.0001	0.04335<br><.0001	0.04819<br><.0001	0.01767<br><.0001	0.09594<br><.0001	-0.01616<br><.0001	-0.04408<br><.0001	0.06045<br><.0001	0.01809<br><.0001	-0.07573<br><.0001	0.03159<br><.0001	-0.00313<br>0.3212	-0.02867<br><.0001
OBESITY	-0.11612<br><.0001	0.03790<br><.0001	-0.01567<br><.0001	0.00736<br>0.0195	0.01702<br><.0001	0.16188<br><.0001	0.08858<br><.0001	1.00000	0.03643<br><.0001	0.01245<br><.0001	0.02658<br><.0001	-0.01705<br><.0001	-0.02042<br><.0001	0.00001<br>0.9982	-0.03313<br><.0001	-0.02285<br><.0001	-0.00924<br>0.0033	0.02357<br><.0001	0.05849<br><.0001	0.03125<br><.0001	-0.01356<br><.0001	-0.01217<br>0.0001	-0.02980<br><.0001	0.01486<br><.0001	-0.09634<br><.0001	-0.05656<br><.0001	0.00444<br>0.1583	-0.00920<br>0.0035
HF<br>HF	0.08614<br><.0001	-0.04384<br><.0001	0.00019<br>0.9524	-0.01199<br>0.0001	-0.42217<br><.0001	0.09702<br><.0001	0.06222<br><.0001	0.03643<br><.0001	1.00000	0.08838<br><.0001	0.14794<br><.0001	0.06936<br><.0001	0.03144<br><.0001	0.01595<br><.0001	0.06409<br><.0001	0.02247<br><.0001	0.03809<br><.0001	0.08106<br><.0001	0.15766<br><.0001	0.23137<br><.0001	0.05044<br><.0001	0.06247<br><.0001	-0.02614<br><.0001	0.03305<br><.0001	0.02732<br><.0001	-0.01024<br>0.0011	-0.00449<br>0.1537	-0.01104<br>0.0005
CIHD	0.11595<br><.0001	-0.00768<br>0.0147	-0.04582<br><.0001	0.00058<br>0.8534	-0.03097<br><.0001	0.02360<br><.0001	0.07380<br><.0001	0.01245<br><.0001	0.08838<br><.0001	1.00000	0.51508<br><.0001	0.05720<br><.0001	0.02821<br><.0001	0.01653<br><.0001	0.03656<br><.0001	0.03060<br><.0001	0.03116<br><.0001	0.03930<br><.0001	0.06120<br><.0001	0.07933<br><.0001	-0.00306<br>0.3320	0.00871<br>0.0057	0.02490<br><.0001	0.01798<br><.0001	-0.00084<br>0.7888	0.00482<br>0.1258	-0.00353<br>0.2628	-0.01296<br><.0001
AF	0.26692<br><.0001	-0.04144<br><.0001	-0.06568<br><.0001	-0.01932<br><.0001	-0.05557<br><.0001	0.02945<br><.0001	0.08730<br><.0001	0.02658<br><.0001	0.14794<br><.0001	0.51508<br><.0001	1.00000	0.06162<br><.0001	0.03691<br><.0001	0.01692<br><.0001	0.05877<br><.0001	0.04559<br><.0001	0.07736<br><.0001	0.06066<br><.0001	0.09293<br><.0001	0.13148<br><.0001	-0.00459<br>0.1455	0.02467<br><.0001	0.03456<br><.0001	0.02317<br><.0001	-0.00945<br>0.0027	0.03967<br><.0001	-0.01549<br><.0001	-0.01587<br><.0001
VHD	0.05392<br><.0001	0.00559<br>0.0759	-0.02267<br><.0001	0.02711<br><.0001	-0.02372<br><.0001	-0.01703<br><.0001	0.06390<br><.0001	-0.01705<br><.0001	0.06936<br><.0001	0.05720<br><.0001	0.06162<br><.0001	1.00000	0.02926<br><.0001	0.00892<br>0.0046	0.02178<br><.0001	0.00906<br>0.0040	0.01850<br><.0001	-0.00115<br>0.7154	0.09467<br><.0001	0.03688<br><.0001	-0.02174<br><.0001	0.01210<br>0.0001	0.02828<br><.0001	0.02389<br><.0001	-0.01710<br><.0001	-0.00277<br>0.3788	-0.00456<br>0.1482	-0.01836<br><.0001
PAD<br>PAD	0.09453<br><.0001	-0.01462<br><.0001	-0.02691<br><.0001	0.05553<br><.0001	0.00252<br>0.4235	-0.00941<br>0.0028	0.07225<br><.0001	-0.02042<br><.0001	0.03144<br><.0001	0.02821<br><.0001	0.03691<br><.0001	0.02926<br><.0001	1.00000	0.01056<br>0.0008	0.06023<br><.0001	0.03660<br><.0001	0.00752<br>0.0170	0.08129<br><.0001	0.02099<br><.0001	0.04907<br><.0001	0.03129<br><.0001	-0.00760<br>0.0158	0.00815<br>0.0096	0.00333<br>0.2906	-0.00194<br>0.5378	0.00829<br>0.0085	0.00273<br>0.3854	-0.01335<br><.0001
PPCI	0.02631<br><.0001	-0.02150<br><.0001	-0.00358<br>0.2560	0.03247<br><.0001	-0.00218<br>0.4894	0.02017<br><.0001	0.04839<br><.0001	0.00001<br>0.9982	0.01595<br><.0001	0.01653<br><.0001	0.01692<br><.0001	0.00892<br>0.0046	0.01056<br>0.0008	1.00000	0.05620<br><.0001	0.02101<br><.0001	0.01675<br><.0001	0.00643<br>0.0412	0.00635<br>0.0437	0.02325<br><.0001	-0.00540<br>0.0866	-0.00061<br>0.8462	0.02457<br><.0001	0.00603<br>0.0556	-0.01864<br><.0001	-0.00446<br>0.1568	-0.00018<br>0.9543	-0.00779<br>0.0134
PCABG	0.12536<br><.0001	-0.09134<br><.0001	-0.02949<br><.0001	0.07794<br><.0001	-0.02170<br><.0001	0.07429<br><.0001	0.13172<br><.0001	-0.03313<br><.0001	0.06409<br><.0001	0.03656<br><.0001	0.05877<br><.0001	0.02178<br><.0001	0.06023<br><.0001	0.05620<br><.0001	1.00000	0.04579<br><.0001	0.06900<br><.0001	0.03291<br><.0001	0.00165<br>0.5994	0.09151<br><.0001	0.00332<br>0.2921	-0.00427<br>0.1756	0.03539<br><.0001	0.00725<br>0.0215	-0.03649<br><.0001	-0.00112<br>0.7222	-0.01208<br>0.0001	-0.00254<br>0.4204
PS	0.06292<br><.0001	0.02028<br><.0001	0.01275<br><.0001	0.04692<br><.0001	0.00532<br>0.0913	0.04203<br><.0001	0.07491<br><.0001	-0.02285<br><.0001	0.02247<br><.0001	0.03060<br><.0001	0.04559<br><.0001	0.00906<br>0.0040	0.03660<br><.0001	0.02101<br><.0001	0.04579<br><.0001	1.00000	0.03849<br><.0001	0.01891<br><.0001	-0.00278<br>0.3777	0.04483<br><.0001	0.01473<br><.0001	-0.01006<br>0.0014	0.02690<br><.0001	0.00349<br>0.2675	-0.01666<br><.0001	0.07185<br><.0001	-0.00094<br>0.7654	-0.00202<br>0.5214
PP	0.10851<br><.0001	0.00639<br>0.0424	-0.01568<br><.0001	0.02189<br><.0001	-0.01214<br>0.0001	0.00764<br>0.0153	0.04335<br><.0001	-0.00924<br>0.0033	0.03809<br><.0001	0.03116<br><.0001	0.07736<br><.0001	0.01850<br><.0001	0.00752<br>0.0170	0.01675<br><.0001	0.06900<br><.0001	0.03849<br><.0001	1.00000	0.01260<br><.0001	0.01065<br>0.0007	0.04445<br><.0001	-0.00580<br>0.0657	-0.00344<br>0.2747	0.03724<br><.0001	0.00264<br>0.4022	-0.02432<br><.0001	0.02331<br><.0001	-0.00733<br>0.0199	0.00645<br>0.0407
COPD	0.12228<br><.0001	0.00457<br>0.1469	-0.08273<br><.0001	0.20585<br><.0001	-0.01994<br><.0001	0.03237<br><.0001	0.04819<br><.0001	0.02357<br><.0001	0.08106<br><.0001	0.03930<br><.0001	0.06066<br><.0001	-0.00115<br>0.7154	0.08129<br><.0001	0.00643<br>0.0412	0.03291<br><.0001	0.01891<br><.0001	0.01260<br><.0001	1.00000	0.06042<br><.0001	0.03387<br><.0001	-0.01494<br><.0001	-0.00120<br>0.7032	0.01535<br><.0001	0.00675<br>0.0322	-0.00916<br>0.0037	0.00702<br>0.0260	0.00760<br>0.0159	0.01446<br><.0001
PHTN	0.00488<br>0.1214	0.03893<br><.0001	0.01363<br><.0001	-0.00408<br>0.1949	-0.09358<br><.0001	0.02306<br><.0001	0.01767<br><.0001	0.05849<br><.0001	0.15766<br><.0001	0.06120<br><.0001	0.09293<br><.0001	0.09467<br><.0001	0.02099<br><.0001	0.00635<br>0.0437	0.00165<br>0.5994	-0.00278<br>0.3777	0.01065<br>0.0007	0.06042<br><.0001	1.00000	0.10762<br><.0001	0.00942<br>0.0028	0.07425<br><.0001	-0.00900<br>0.0043	0.04714<br><.0001	-0.00093<br>0.7685	-0.02258<br><.0001	-0.00548<br>0.0821	-0.02905<br><.0001
ckd<br>ckd	0.16360<br><.0001	-0.07299<br><.0001	0.03716<br><.0001	-0.03668<br><.0001	-0.32162<br><.0001	0.19921<br><.0001	0.09594<br><.0001	0.03125<br><.0001	0.23137<br><.0001	0.07933<br><.0001	0.13148<br><.0001	0.03688<br><.0001	0.04907<br><.0001	0.02325<br><.0001	0.09151<br><.0001	0.04483<br><.0001	0.04445<br><.0001	0.03387<br><.0001	0.10762<br><.0001	1.00000	0.35533<br><.0001	0.06725<br><.0001	0.00733<br>0.0199	0.04448<br><.0001	0.00281<br>0.3720	0.01647<br><.0001	-0.01351<br><.0001	-0.01036<br>0.0010
ESRD	-0.00917<br>0.0036	-0.00335<br>0.2874	0.07852<br><.0001	-0.06161<br><.0001	-0.11655<br><.0001	0.11999<br><.0001	-0.01616<br><.0001	-0.01356<br><.0001	0.05044<br><.0001	-0.00306<br>0.3320	-0.00459<br>0.1455	-0.02174<br><.0001	0.03129<br><.0001	-0.00540<br>0.0866	0.00332<br>0.2921	0.01473<br><.0001	-0.00580<br>0.0657	-0.01494<br><.0001	0.00942<br>0.0028	0.35533<br><.0001	1.00000	0.00962<br>0.0023	-0.01830<br><.0001	-0.01187<br>0.0002	0.03972<br><.0001	-0.01222<br>0.0001	-0.00713<br>0.0237	-0.00822<br>0.0091
LC	-0.07838<br><.0001	-0.04128<br><.0001	0.02866<br><.0001	0.01923<br><.0001	-0.05894<br><.0001	-0.00740<br>0.0188	-0.04408<br><.0001	-0.01217<br>0.0001	0.06247<br><.0001	0.00871<br>0.0057	0.02467<br><.0001	0.01210<br>0.0001	-0.00760<br>0.0158	-0.00061<br>0.8462	-0.00427<br>0.1756	-0.01006<br>0.0014	-0.00344<br>0.2747	-0.00120<br>0.7032	0.07425<br><.0001	0.06725<br><.0001	0.00962<br>0.0023	1.00000	-0.02317<br><.0001	0.04462<br><.0001	0.01385<br><.0001	-0.02617<br><.0001	-0.00302<br>0.3376	0.00160<br>0.6115
HMALIG	0.13331<br><.0001	0.02721<br><.0001	-0.05469<br><.0001	0.06047<br><.0001	0.02963<br><.0001	-0.01633<br><.0001	0.06045<br><.0001	-0.02980<br><.0001	-0.02614<br><.0001	0.02490<br><.0001	0.03456<br><.0001	0.02828<br><.0001	0.00815<br>0.0096	0.02457<br><.0001	0.03539<br><.0001	0.02690<br><.0001	0.03724<br><.0001	0.01535<br><.0001	-0.00900<br>0.0043	0.00733<br>0.0199	-0.01830<br><.0001	-0.02317<br><.0001	1.00000	0.00309<br>0.3266	-0.01257<br><.0001	0.01991<br><.0001	-0.00850<br>0.0069	-0.00516<br>0.1017
DA	-0.00571<br>0.0699	0.02714<br><.0001	0.01813<br><.0001	0.00802<br>0.0109	-0.02540<br><.0001	0.01647<br><.0001	0.01809<br><.0001	0.01486<br><.0001	0.03305<br><.0001	0.01798<br><.0001	0.02317<br><.0001	0.02389<br><.0001	0.00333<br>0.2906	0.00603<br>0.0556	0.00725<br>0.0215	0.00349<br>0.2675	0.00264<br>0.4022	0.00675<br>0.0322	0.04714<br><.0001	0.04448<br><.0001	-0.01187<br>0.0002	0.04462<br><.0001	0.00309<br>0.3266	1.00000	0.02764<br><.0001	0.00789<br>0.0122	0.00204<br>0.5182	0.00285<br>0.3655
MAL	0.01463<br><.0001	0.00172<br>0.5849	0.01644<br><.0001	-0.06718<br><.0001	-0.05817<br><.0001	-0.05579<br><.0001	-0.07573<br><.0001	-0.09634<br><.0001	0.02732<br><.0001	-0.00084<br>0.7888	-0.00945<br>0.0027	-0.01710<br><.0001	-0.00194<br>0.5378	-0.01864<br><.0001	-0.03649<br><.0001	-0.01666<br><.0001	-0.02432<br><.0001	-0.00916<br>0.0037	-0.00093<br>0.7685	0.00281<br>0.3720	0.03972<br><.0001	0.01385<br><.0001	-0.01257<br><.0001	0.02764<br><.0001	1.00000	0.02159<br><.0001	0.01095<br>0.0005	-0.00486<br>0.1230
DEM	0.22282<br><.0001	0.05301<br><.0001	0.00311<br>0.3236	-0.03832<br><.0001	0.01699<br><.0001	0.01701<br><.0001	0.03159<br><.0001	-0.05656<br><.0001	-0.01024<br>0.0011	0.00482<br>0.1258	0.03967<br><.0001	-0.00277<br>0.3788	0.00829<br>0.0085	-0.00446<br>0.1568	-0.00112<br>0.7222	0.07185<br><.0001	0.02331<br><.0001	0.00702<br>0.0260	-0.02258<br><.0001	0.01647<br><.0001	-0.01222<br>0.0001	-0.02617<br><.0001	0.01991<br><.0001	0.00789<br>0.0122	0.02159<br><.0001	1.00000	0.00001<br>0.9968	0.01846<br><.0001
MDD	-0.03264<br><.0001	0.02745<br><.0001	-0.01610<br><.0001	0.00009<br>0.9775	-0.00243<br>0.4409	-0.00713<br>0.0237	-0.00313<br>0.3212	0.00444<br>0.1583	-0.00449<br>0.1537	-0.00353<br>0.2628	-0.01549<br><.0001	-0.00456<br>0.1482	0.00273<br>0.3854	-0.00018<br>0.9543	-0.01208<br>0.0001	-0.00094<br>0.7654	-0.00733<br>0.0199	0.00760<br>0.0159	-0.00548<br>0.0821	-0.01351<br><.0001	-0.00713<br>0.0237	-0.00302<br>0.3376	-0.00850<br>0.0069	0.00204<br>0.5182	0.01095<br>0.0005	0.00001<br>0.9968	1.00000	0.00138<br>0.6609
AWEEKEND<br>Admission day is a weekend	-0.00133<br>0.6724	0.01080<br>0.0006	0.00939<br>0.0029	-0.00142<br>0.6513	-0.00573<br>0.0687	-0.00827<br>0.0087	-0.02867<br><.0001	-0.00920<br>0.0035	-0.01104<br>0.0005	-0.01296<br><.0001	-0.01587<br><.0001	-0.01836<br><.0001	-0.01335<br><.0001	-0.00779<br>0.0134	-0.00254<br>0.4204	-0.00202<br>0.5214	0.00645<br>0.0407	0.01446<br><.0001	-0.02905<br><.0001	-0.01036<br>0.0010	-0.00822<br>0.0091	0.00160<br>0.6115	-0.00516<br>0.1017	0.00285<br>0.3655	-0.00486<br>0.1230	0.01846<br><.0001	0.00138<br>0.6609	1.00000

Table S4. Subgroup analysis according to younger (age <65 years) and older (≥65 years) patients

Age Group	Outcome	Frailty (+)	Frailty (-)	Adjusted Odds Ratio[a]	*P*-value
**Age <65 years** (N=216,375)	Sample size	166,390	49,985	-	-
	In-hospital mortality (%)	30.6	15.1	2.56 (2.40-2.73)	<.001
	Do-not resuscitate (%)	21.2	9.6	2.47 (2.28-2.66)	<.001
	Palliative care consult (%)	18.3	8.7	2.23 (2.06-2.42)	<.001
	Skilled nursing facility (%)	19.7	7.7	2.52 (2.32-2.74)	<.001
	Revascularization (%)	4.9	11.9	0.39 (0.36-0.43)	<.001
	MCS (%)	15.8	15.6	1.05 (0.97-1.12)	0.229
	Heart transplant (%)	2.6	2.5	1.01 (0.84-1.21)	0.930
	Intracranial hemorrhage (%)	2.1	0.8	2.66 (2.09-3.38)	<.001
	Gastrointestinal hemorrhage (%)	6.9	2.1	3.25 (2.79-3.78)	<.001
	Acute kidney injury (%)	73.4	29.4	8.52 (8.07-9.00)	<.001
	Delirium (%)	4.8	0.3	17.64 (12.04-25.84)	<.001
	Length of stay (days ± SD)	15.7 ± 18.9	9.5 ± 12.2	4.75 (4.37-5.13)[b]	<.001
	Total hospital cost ($ ± SD)	76,838 ± 110,252	419,192 ± 71,225	21,963 (19,740-24,187)[b]	<.001
**Age ≥65 years** (N=287,405)	Sample size	237,200	50,205	-	-
	In-hospital mortality (%)	38.3	25.7	1.80 (1.71-1.89)	<.001
	Do-not resuscitate (%)	36.4	22.8	1.77 (1.68-1.87)	<.001
	Palliative care consult (%)	26.7	15.5	1.83 (1.72-1.94)	<.001
	Skilled nursing facility (%)	30.8	18.1	1.83 (1.73-1.94)	<.001
	Revascularization (%)	7.5	16.9	0.48 (0.45-0.52)	<.001
	MCS (%)	8.0	11.2	0.80 (0.73-0.86)	<.001
	Heart transplant (%)	0.5	0.6	0.99 (0.71-1.38)	0.954
	Intracranial hemorrhage (%)	1.4	0.3	4.34 (3.07-6.15)	<.001
	Gastrointestinal hemorrhage (%)	6.7	2.6	2.54 (2.23-2.89)	<.001
	Acute kidney injury (%)	72.8	29.0	7.81 (7.41-8.23)	<.001
	Delirium (%)	6.5	0.5	13.35 (10.13-17.61)	<.001
	Length of stay (days ± SD)	11.4 ± 12.5	7.3 ± 7.2	3.60 (3.35-3.84)[b]	<.001
	Total hospital cost ($ ± SD)	49,510 ± 65,955	36,421 ± 42,995	13,153 (11,844-14,461)[b]	<.001

[a]Adjusted for age, sex, smoking, diabetes mellitus, hyperlipidemia, obesity, heart failure, chronic ischemic heart disease, valvular heart disease, previous PCI, previous CABG, previous stroke, previous pacemaker, chronic obstructive pulmonary disease, pulmonary hypertension, end-stage renal disease, deficiency anemia, malnutrition, major depression, and weekend admission

[b]Adjusted mean difference with 95% confidence interval

Abbreviations: CABG, coronary artery bypass graft; MCS, mechanical circulatory support; SD, standard deviation
